# The Integration and Growth of R in Soil Research: A 10‐Year Analysis

**DOI:** 10.1002/ece3.71545

**Published:** 2025-06-26

**Authors:** Meixiang Gao, Xiujuan Yan, Xin Li, Ye Zheng, Jiangshan Lai

**Affiliations:** ^1^ Department of Geography and Spatial Information Techniques Ningbo University Ningbo China; ^2^ Zhejiang Collaborative Innovation Center for Land and Marine Spatial Utilization and Governance Research Ningbo University Ningbo China; ^3^ Jilin Academy of Agricultural Sciences Changchun China; ^4^ Faculty of Electrical Engineering and Computer Science Ningbo University Ningbo China; ^5^ College of Ecology and Environment Nanjing Forestry University Nanjing China

**Keywords:** R packages, R software, soil ecology, soil journal, soil science

## Abstract

The field of soil science has seen significant advancements in recent years, largely due to the integration of computational tools and statistical methods. Among these resources, the programming language R has emerged as a powerful and versatile platform for soil scientists, aiding in a spectrum of tasks from data analysis and modeling to visualization. Nonetheless, the broader trends and specific patterns of R usage in soil research have not been thoroughly documented. Our study investigated the prevalence of R and its package usage in 25,888 research articles published in 10 leading soil science journals over a decade, from 2014 to 2023. A considerable number of these articles, 7899 (or 30.5%), named R as their primary data analysis tool. The use of R has followed a steady linear growth pattern, rising from 13.9% in 2014 to 46.5% in 2023. The most commonly used R packages were “vegan,” “ggplot2,” “lme4,” “nlme,” and “randomForest,” with each journal showcasing unique research focuses, resulting in varying frequencies of R package applications across different publications. Furthermore, there was a notable increase in the average number of R packages used per article throughout the study period. This research highlights the pivotal role of R, armed with its robust statistical and visualization capabilities, in enabling soil scientists to conduct comprehensive analyses and gain in‐depth insights into the complex dimensions of soil science.

## Graphical abstract

Our study provides a comprehensive analysis of the adoption and usage patterns of the R programming language in soil science research over the past decade. R usage in soil research grew linearly from 13.9% to 64.5% in top journals from 2014 to 2023.

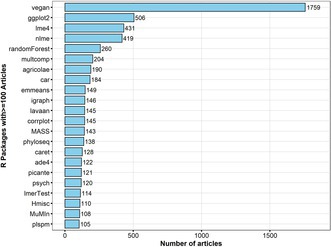

## 1 Introduction

As the foundation of supporting terrestrial life on the planet (Bardgett and Van Der Putten 2014), soils serve as home to diverse organisms and provide various ecosystem services (Guerra et al. 2022; Outhwaite et al. 2022), such as nutrient cycling, carbon sequestration, water filtration, and pest control, which are critical for food supply and human well‐being (Wall et al. 2015). Soil science plays a crucial role in understanding the complex interactions between soil ecosystems and their surrounding environments (Xu 2022). The study of soils employs a multifaceted approach, integrating disciplines, such as biology, chemistry, physics, and ecology, to probe the intricate interactions that occur within soil ecosystems and assess their effects on the wider environment (Xu 2022). In recent years, the growing importance of soil science has been increasingly recognized, especially against the backdrop of worldwide issues such as climate change, the struggle for food security, and the imperative of environmental sustainability (Guerra et al. 2022).

Acknowledging the intricate nature of soils and their underlying processes, soil ecologists are increasingly embracing mathematical and statistical methodologies to address scientific inquiries and simulate spatial and temporal dynamics (Webster 2001; Potapov et al. 2023). The diverse array of soil data reflects the complexity of soil systems, encapsulating interactions among biological, chemical, hydrological, and biophysical processes (Todd‐Brown et al. 2022). These data are indispensable for tackling pivotal scientific challenges, including biodiversity conservation, biogeochemical process studies, soil taxonomy, agronomy, and micrometeorology. However, soil researchers now confront significant obstacles in data analysis, exacerbated by the increasing quality, volume, and frequency of data in the era of big data (Vestergaard et al. 2017; Liu et al. 2024). Consequently, there is an urgent need for sophisticated methodologies to dissect vast datasets and model the dynamic processes occurring within soils (Peng et al. 2022). Although traditional approaches remain valuable, they often struggle to address the numerous soil and related environmental variables and the multifaceted nature of soil ecosystems. This has driven a shift towards more advanced computational techniques that are better equipped to handle the complexity and scale of modern soil science research (Todd‐Brown et al. 2022).

The swift evolution of computational methodologies has prompted soil researchers to adopt programming languages and statistical packages for enhanced analytical precision and depth (Webster 2001). These tools allow for the management and interpretation of large datasets, the development of predictive models, and the simulation of various soil processes under different environmental conditions (Vestergaard et al. 2017; Zhang et al. 2023). Among the array of available tools, the R programming language has emerged as a particularly powerful and versatile option for soil scientists.

R is an open‐source programming language that was developed to merge the useful features of the S and Scheme programming languages (Ihaka and Gentleman 1996). R provides a broad spectrum of statistical and graphical techniques (Grunsky 2002), making it an ideal tool for soil science research (Sousa et al. 2020). Its flexibility allows researchers to customize analyses to fit the specific needs of soil studies, whether it be spatial analysis (Yang et al. 2023), multivariate statistics (Outhwaite et al. 2022), or time series modeling (Bean et al. 2018).

R boasts an extensive and varied collection of packages, a system that is collaboratively developed and enhanced by a global community of developers. These packages cover nearly every aspect of data analysis methods that may be needed in the field of soil science, as well as specialized packages tailored for soil research (Moro Rosso et al. 2021), providing researchers with powerful tool support. This makes the application of R extremely extensive and profound in the field of soil scientific research (Sousa et al. 2020).

Despite the growing application of the R programming language in soil science, there is currently a lack of systematic analysis regarding its usage trends and patterns within the field. This study aims to address this gap by conducting a quantitative analysis of research articles published in main soil science journals from 2014 to 2023, focusing on the utilization of R and its associated packages. The primary research questions of this study include: (1) How prevalent is the use of R in soil science research? (2) Which R packages are most commonly employed in soil science studies? (3) Are there differences in the usage of R packages across different soil science journals? (4) How have the trends and patterns of R package usage evolved over time?

Through the exploration of these queries, the objectives of this study are to deliver an extensive perception of the incorporation of R in soil scientific inquiry. The results are poised not only to underscore the prevailing utilization of R within the discipline but also to shed light on the instruments and techniques that have become essential to contemporary soil research. Furthermore, discerning the usage patterns and trends of R can guide the trajectory of future scholarly endeavors and pinpoint opportunities for enhancing or broadening the application of R. This investigation marks a pivotal phase in acknowledging the contribution of R to the progression of soil science, emphasizing the necessity for ongoing development in data interpretation and model construction within this field.

## 2 Materials and Methods

In order to comprehensively assess the usage of R and its packages in soil science studies, we selected a range of prominent journals that are highly regarded in the field. Our criteria for selection were based on journals that had an impact factor greater than 3.0 as of 2022, as measured by the Web of Science (WoS) Journal Citation Reports (JCR) under the “soil” category, accessible at www.webofknowledge.com. Additionally, we omitted journals that have published less than 50 papers per year since 2014 to ensure a statistically significant sample size. The stringent selection process identified the following esteemed journals: “Applied Soil Ecology” (ASE), “Biology and Fertility of Soils” (BFS), “Catena”, “European Journal of Soil Biology” (EJSB), “European Journal of Soil Science” (EJSS), “Geoderma,” “Pedosphere,” “Plant and Soil” (PS), “Soil and Tillage Research” (STR), and “Soil Biology and Biochemistry” (SBB). Subsequently, in this study, these publications were designated as the “ten leading soil science journals.”

We acknowledged that some articles might mention the use of R or R packages in the “Methods” section without explicitly citing them in the “References.” To ensure the accuracy and comprehensiveness of our analysis, we conducted a detailed manual review of the “Methods” sections within our collection of papers. This diligent approach allowed us to identify and include all instances where R or R packages were utilized, even when they were not directly mentioned in the “References” section. During the review process, we recorded the names of all R packages that were employed. This strategy guarantees that our findings accurately reflect the importance of R and its related packages in the field of soil research.

The base packages of R, such as “base,” “stats,” and “graphics,” were omitted from the analysis. This decision was made because these packages offer essential support functions for R programming, and their inclusion would not align with the goals of the current study. Every computation within this manuscript was performed utilizing the R 4.4.1 statistical programming language (R Core Team 2024). The data (R file) and the code for this study are provided in Appendix S1, allowing readers to readily generate the figures presented.

## 3 Results

### 3.1 Trends in the Utilization of R

With careful dedication, we assembled an extensive dataset consisting of 25,888 scholarly articles, drawn from 10 chosen soil science journals, spanning a decade from 2014 to 2023.

**FIGURE 1:**
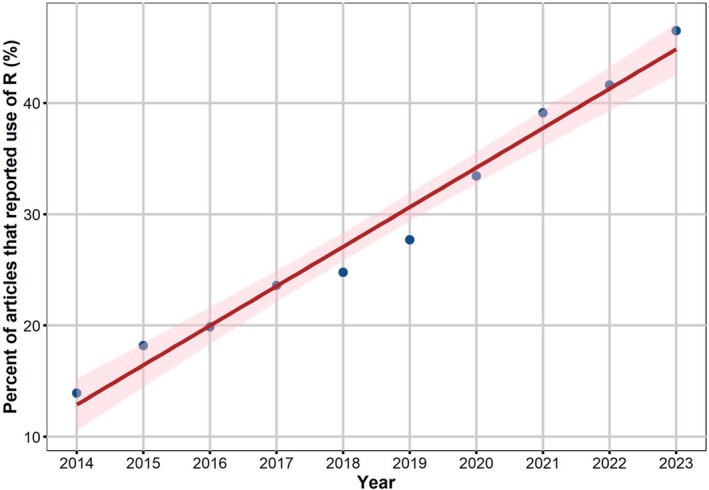
The annual proportion of scholarly articles employing R in the 10 leading soil journals from 2014 to 2023. Data compiled from more than 25,888 articles, with a coefficient of *r* = 0.98 (*p* < 0.001). The percentage was computed by dividing the number of research articles that utilized R by the total number of research articles published in a specific year.

Within this collection of articles, 7899 papers, constituting around 30.5% of the entire dataset, specifically cited R as the statistical software employed for data analysis. This result highlights the extensive integration of R in soil science research. Across the years, the proportion of articles documenting the use of R has progressively risen, from 13.9% in 2014 to 46.5% in 2023 (Figure 1). Moreover, the annual rate of R usage showed upward trend over time (*r* = 0.98, *p* < 0.001), indicating a consistent increase in adoption across the study period.

**FIGURE 2:**
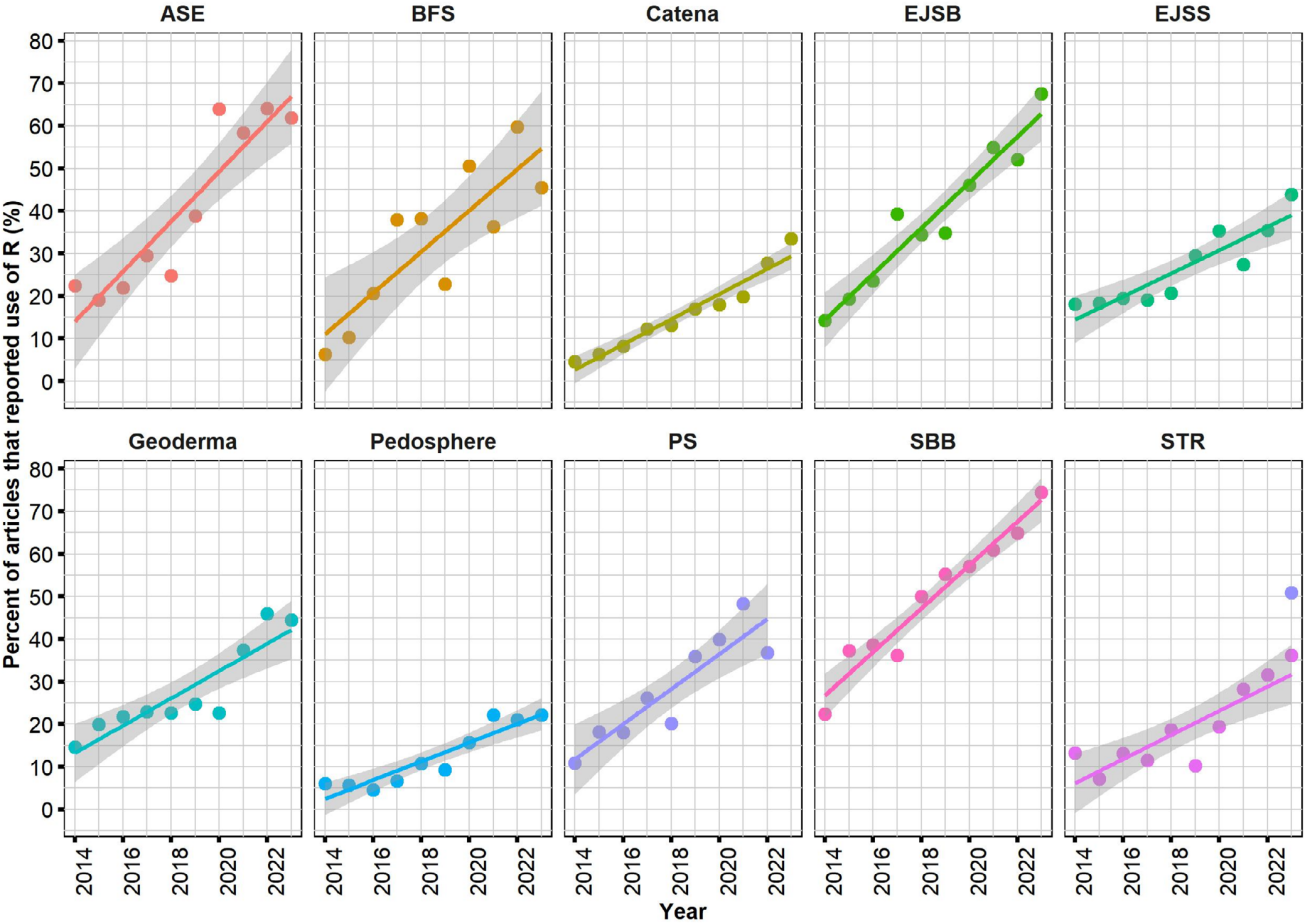
Trends in the proportion of scholarly articles employing R in the 10 leading soil science journals (2014–2023). The graph illustrates the annual percentage of research articles explicitly citing the use of R in the following leading soil science journals: “Applied Soil Ecology” (ASE), “Biology and Fertility of Soils” (BFS), “Catena”, “European Journal of Soil Biology” (EJSB), “European Journal of Soil Science” (EJSS), “Geoderma”, “Pedosphere”, “Plant and Soil” (PS), “Soil Biology and Biochemistry” (SBB), and “Soil and Tillage Research” (STR).

Throughout our comprehensive study, which spanned from 2014 to 2023, a clear upward trend was noted in the proportion of articles that utilized R for data analysis across the selected journals (Figure 2). However, it is noteworthy to point out that there were considerable fluctuations among certain journals, and the rate of growth in R usage differed across the various publications (Figure 2, Table 1).

**TABLE 1:** R usage prevalence and growth trends by journal.

Journal name	Total articles published	Articles using R	R usage prevalence (%)	Intercept (baseline)	Annual growth rate (%)
ASE	2824	1242	44	−118.04	5.9
BFS	897	283	31.5	−97.51	4.8
Catena	5021	965	19.2	−59.62	2.9
EJSB	675	257	38.1	−108.05	5.3
EJSS	1075	298	27.7	−54.66	2.7
Geoderma	4647	1337	28.8	−64.53	3.2
Pedosphere	867	107	12.3	−44.42	2.2
PS	4429	1421	32.1	−85.08	4.2
SBB	3123	1515	48.5	−102.42	5.1
STR	2330	474	20.3	−57.08	2.8

*Note:* Intercept (baseline): Predicted R usage prevalence (%) at the baseline year (year = 0 in the regression model). Annual growth rate: Slope of the linear regression model, indicating the annual percentage change in R usage prevalence.

The journal SBB stood out for its strong dedication to R, achieving an impressive adoption rate of 48.5% over the decade. Remarkably, as early as 2014, SBB had recorded a substantial R usage rate of 22.4%, which then saw an average annual growth of 5.1%. By 2023, the fraction of R usage within SBB had surged to a striking 74.5%, highlighting a consistent and strong preference for R within this journal (Figure 2, Table 1).

Coming in as a close second was ASE, which sustained an R usage rate of 44%. Notably, ASE exhibited the fastest rise in R usage among the journals surveyed, boasting a substantial average annual growth rate of 5.9%. The journal saw a significant surge in R usage from 22.4% in 2014 to a remarkable 64.0% by 2022. Despite a slight dip in 2023 to 61.8%, this suggests that the R adoption may be approaching a plateau, indicating a persistent preference for R in this publication (Figure 2, Table 1).

The EJSB started the decade in 2014 with a 14.3% R usage rate, showing an average annual growth rate of 5.3%. By 2023, the R usage rate in the EJSB had risen to 67.5%. Consequently, the journal secured the third position in the overall 10‐year average R usage ratio, with a score of 38.1% (Figure 2, Table 1).

By contrast, PS, BFS, “Geoderma,” and the EJSS showed moderate 10‐year average R usage rates of 32.1%, 31.5%, 28.8%, and 27.7%, respectively. Meanwhile, STR, “Catena,” and “Pedosphere” exhibited comparatively lower R language usage with 10‐year averages of 20.3%, 19.2%, and 12.3%, respectively. It is noteworthy that these three journals experienced a slower growth rate of 2.8%, 2.9%, and 2.2%, respectively (Figure 2, Table 1).

### 3.2 Patterns of R Package Utilization

**FIGURE 3:**
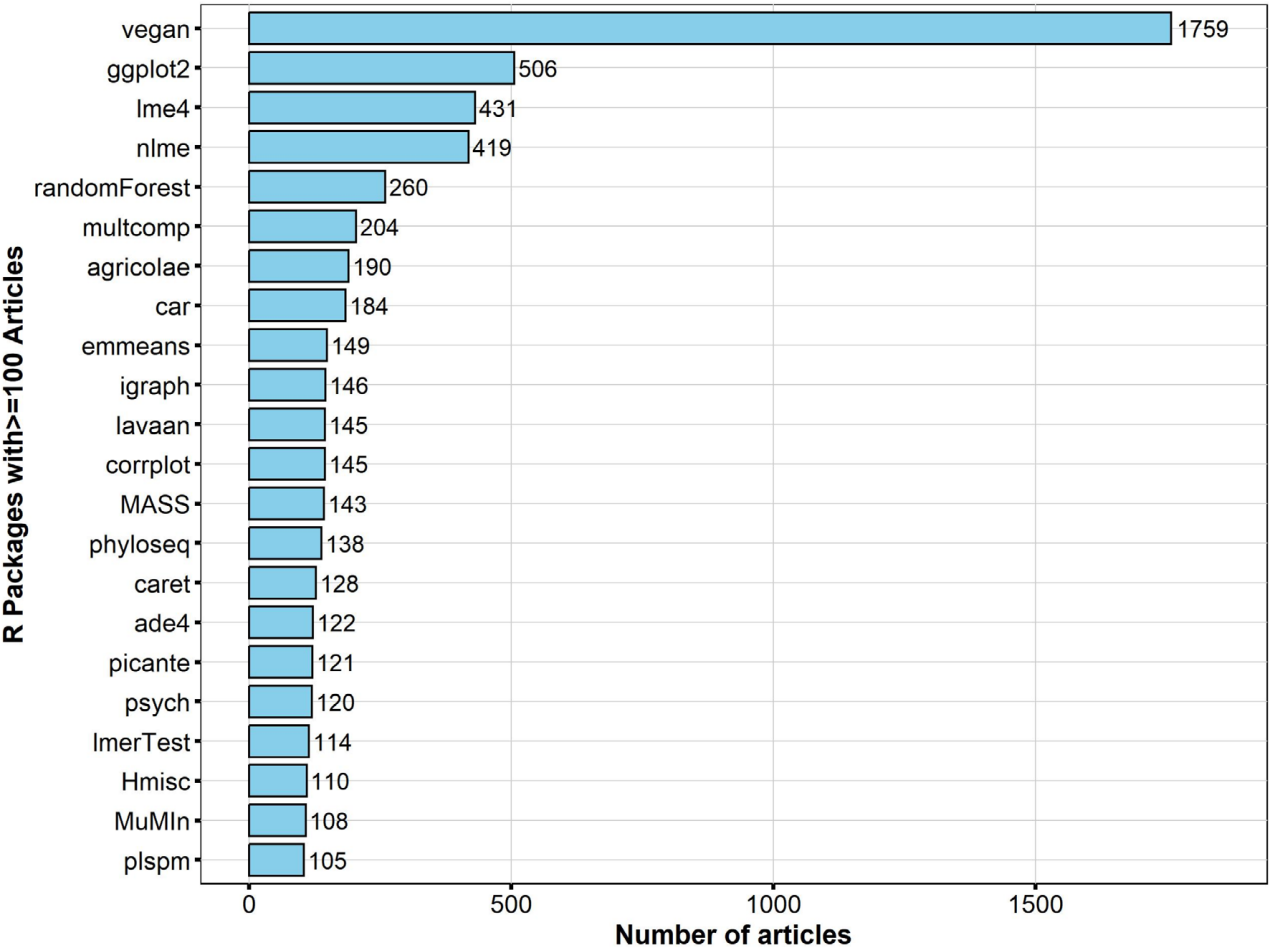
Illustration of the most frequently referenced R packages (appearing in over 100 articles) across the 10 leading soil science journals from 2014 to 2023. These references were identified through an analysis of the methodologies outlined in over 25,888 research articles published in these journals.

Throughout our extensive examination of research articles, we documented the use of a wide variety of R packages, totaling over 1452, which researchers utilized to enhance their data analysis processes. Significantly, 22 of these packages stood out as popular selections, appearing in over 100 articles (Figure 3).

The “vegan” package (Oksanen et al. 2024) stood out at the top of the list, a multifaceted and widely respected tool mainly applied for multivariate analysis in community ecology. Its usage in soil science journals was predominant, exceeding the frequency of the second‐place package by over three times. In the second position was the “ggplot2” package (Wickham 2016), celebrated for its critical role in improving data visualization. The “lme4” package (Bates et al. 2015) ranked third, noted for its powerful capabilities in fitting and analyzing linear mixed models. The “nlme” package (Pinheiro et al. 2020) followed in fourth place, offering flexibility in modeling linear and nonlinear mixed models. Occupying the fifth slot was “randomForest” (Liaw and Wiener 2002), a widely used and potent tool for classification, regression, and various machine learning tasks. Completing the top 10 were “multcomp” (Hothorn et al. 2008), “agricolae” (Mendiburu 2023), “car” (Fox and Weisberg 2019), “emmeans” (Lenth 2023), and “igraph” (Csardi and Nepusz 2006; Csárdi et al. 2024) (Figure 3). For a comprehensive listing of the 22 most commonly utilized packages, please refer to Table S1.

**FIGURE 4:**
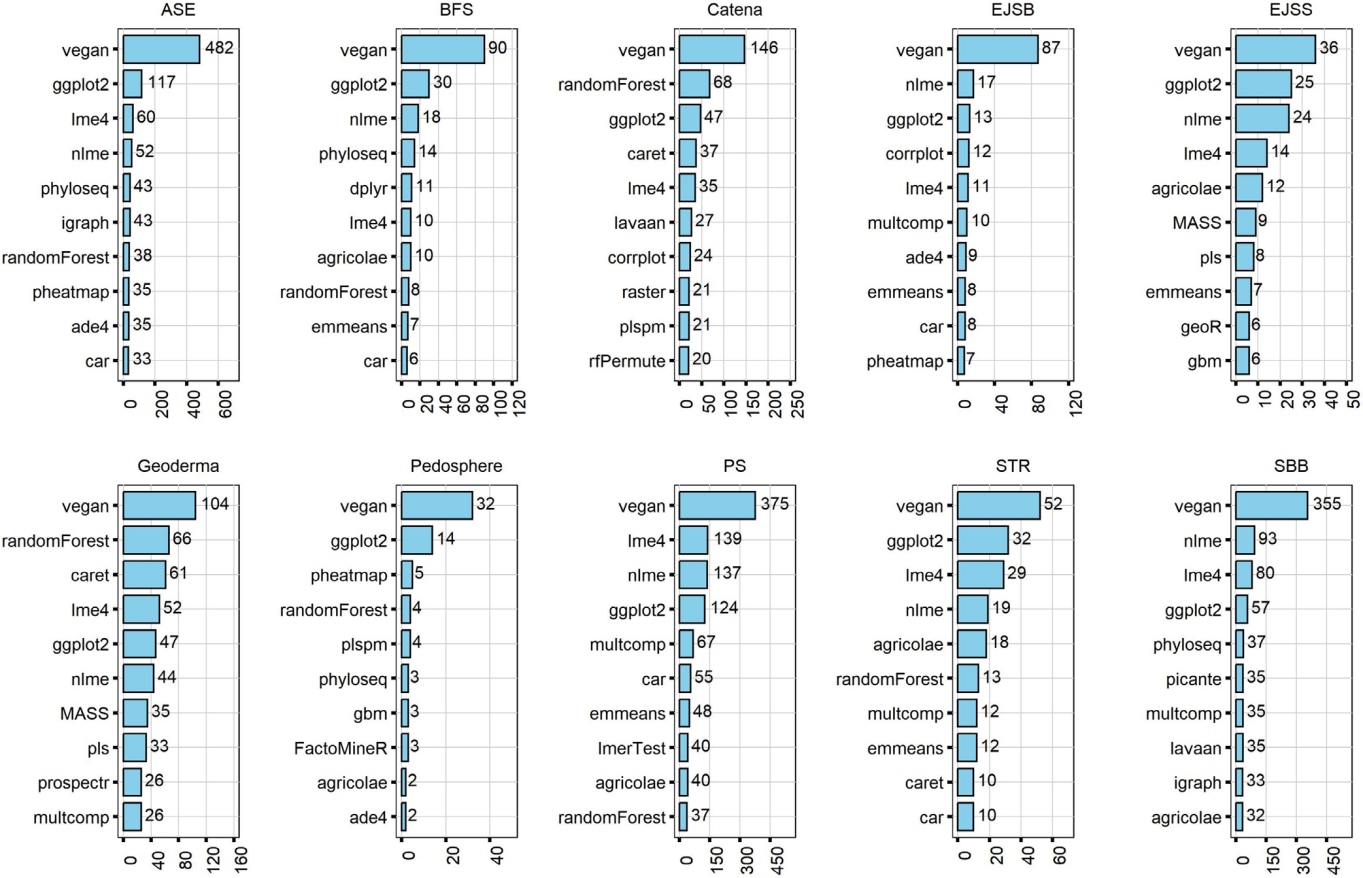
This examination encompasses references to the top 10 frequently cited R packages within each soil journal, spanning from 2014 to 2023. These references were identified through a thorough review of the methodologies presented in more than 25,888 research articles across these journals.

The variety of research topics covered by the different journals has naturally resulted in the use of diverse sets of R packages, as illustrated in Figure 4. The “vegan” package stands out as the favored choice across the 10 journals, solidifying its position as the go‐to package for soil science data analysis (Figure 3). Given its status as the most widely used R package for graphing, it is unsurprising that “ggplot2” package ranks second in terms of frequency of use in five journals (ASE, BFS, EJSS, “Pedosphere,” and STR), resulting in its overall second‐place ranking. Furthermore, mixed‐effect models are prevalent in soil science, as evidenced by the popularity of the “lme4” and “nlme” packages in seven journals (ASE, BFS, EJSB, EJSS, PS, STR, and SBB). Conversely, “randomForest” may hold greater appeal in two journals: “Catena” and “Geoderma,” where the “randomForest” package is ranked second in both.

**FIGURE 5:**
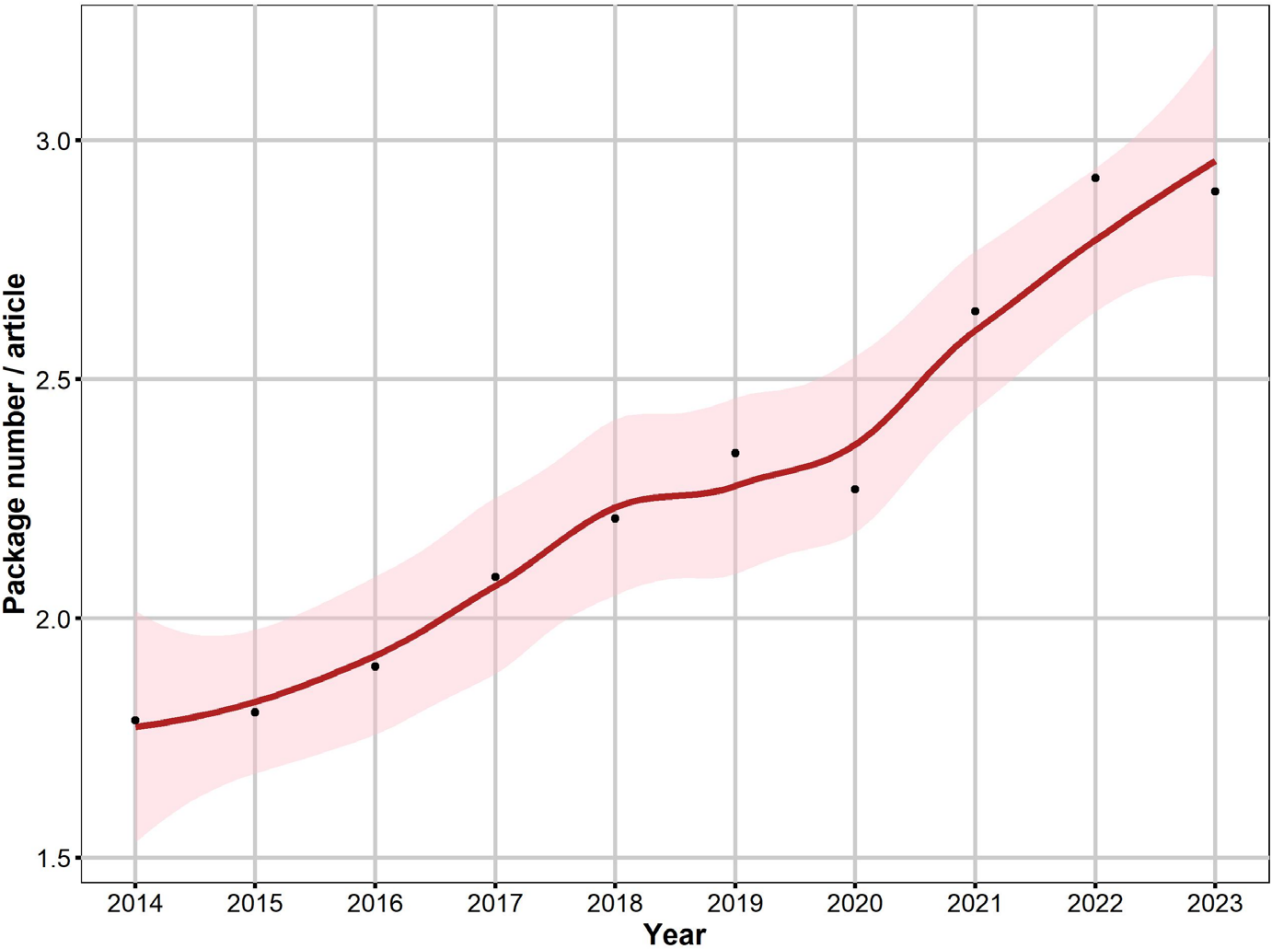
Average number of packages used per article in the 10 leading soil journals from 2014 to 2023.

An interesting trend is the noticeable increase in the average number of R packages used per article (Figure 5). This trend indicates a gradual deepening of sophistication in data analysis approaches within the field of soil studies, marked by an increased reliance on a larger number of R packages per paper. Furthermore, this phenomenon can be attributed to the expanding number of R packages available. The increasing abundance of these tools enhances researchers' capabilities, providing a diverse range of resources for conducting more complex and sophisticated analyses in the realm of soil studies. This growing availability of R packages equips researchers with a more comprehensive set of tools, enabling them to explore and interpret data in increasingly subtle and advanced ways within the context of soil research.

## 4 Discussion

### 4.1 The Increasing Dominance of R in Soil Science Research

Recently, the progress in data‐collecting technologies has resulted in the assembly of vast data collections within the field of soil science (Todd‐Brown et al. 2022). The management and examination of these substantial data volumes have become standard procedures for modern soil science investigators (Curzio et al. 2021). Across soil investigation disciplines, a multitude of statistical analysis program applications are widely applied, each possessing its particular set of benefits and restrictions (Adi and Grunwald 2020; Liu et al. 2024).

Significantly, the current study reveals a marked rise in the prevalence of R as the principal statistical software in scholarly articles featured in the 10 leading soil science journals. Our analysis indicates that the use of R has undergone a significant uptick, escalating from 13.9% in 2014 to 46.5% by 2023. Undeniably, this sharp rise highlights the escalating dominance of the R programming language as the leading data analysis instrument in modern soil research. This pattern of usage corresponds to bibliometric analyses in similar scientific areas, such as ecology (Lai et al. 2019; Gao et al. 2025), forestry (Lai, Zhu, et al. 2023), and conservation biology (Lai, Cui, et al. 2023).

Given the scholarly preference for incorporating cutting‐edge methodologies (Muenchen 2023), the software commonly used in soil science research can serve as leading indicators of the trajectory of the most effective and powerful statistical methods, as well as the direction of data science software development. To quantitatively support this trend, we applied a correlation analysis between year and R usage rate. Although we recognize that correlation with time does not imply causation, the result (*r* = 0.98, *p* < 0.001) supports the observation of a consistent and steady growth trend in R adoption across the examined period.

### 4.2 The Advantages and Applications of R

It is evident that R's strengths, such as its powerful statistical functions, sophisticated data visualization modules, extensive community engagement, open‐source identity, user‐friendly accessibility, and a commendable programming ecosystem that interfaces with code from foreign languages (C, C++, Fortran, etc.) (Dalgaard 2002; Schlather and Huwe 2004), make it an extraordinarily appealing and cutting‐edge option for data analysis and scholarly study in the domain of soil science (Too et al. 2022).

In addition to the R program, other computer programs serve as valuable tools that are also utilized in soil science to address significant scientific questions across a variety of research areas, such as SAS (Afzalinia and Zabihi 2014), SPSS (Zeng et al. 2013), MATLAB (Karkush et al. 2020), GS+ (Penížek and Borůvka 2004), ArcGIS (Bayat et al. 2019), and Python (Przeździecki et al. 2023). However, the present study was limited to evaluating the prevalence of R usage in soil research, precluding the quantification of trends and patterns associated with other computer programs. A thorough examination across a spectrum of academic journals suggests a substantial rise in the utilization of R, accompanied by a decline in the reliance on proprietary software such as SAS, SPSS, and MATLAB (Muenchen 2023). Furthermore, it is likely that researchers have increasingly lessened their reliance on costly commercial software, transitioning to cost‐free, open‐source options such as R and Python, as suggested by Tippmann (2015).

Although we did not evaluate Python's popularity in articles of soil research, our data revealed that R holds dominance in the 10 main soil journals. One of the key reasons for R's prominence in this domain is its capacity to offer highly specialized libraries tailored for soil field analysis, such as “soilphysic” (Silva and Lima 2015), “XPolaris” (Moro Rosso et al. 2021), “ausplotsR” (Munroe et al. 2021), “Soil‐app” (Matias et al. 2021), “SoilR” (Sierra et al. 2012), “soiltestcorr” (Correndo et al. 2023), “microgeo” (Li et al. 2024), and “SimET” (Liu et al. 2023), which are meticulously designed for soil field analysis (Table S2).

### 4.3 Journal‐Level Differences in R Usage

The widespread use of the R programming language in esteemed journals like SBB and ASE, both prominent among the 10 leading soil science journals, is largely due to their close alignment with the field of soil ecological research. The specialized emphasis on soil ecology within these journals inherently demands sophisticated data analysis tools like R to manage the complex soil science data frequently encountered in this field. Consequently, these journals have instinctively adopted R as their leading statistical tool.

On the other hand, the comparatively limited use of R in journals such as STR, “Catena,” and “Pedosphere” can be attributed to their primary focus on classical soil science. As a field, soil science has traditionally laid greater emphasis on conventional approaches, which may not call for the elaborate, data‐driven analyses that R is particularly adept at handling. This distinction in research priorities has resulted in a less common integration of R in these journal's publications.

The preferences of soil scientists for analytical software can also impact the adoption of R software across different journals. However, the observations underscore the diverse and strategic choices made by researchers in different fields and across various journals when it comes to selecting R packages. We emphasize that these journals were selected as representative platforms in the field, and their inclusion does not imply a hierarchical ranking of quality.

### 4.4 The Role of R Packages and Open Science in Advancing Soil Research

The formidable statistical prowess of R is significantly bolstered by its vast repository of packages. These packages are often developed by specialists in computation, coders, and engaged users, including geoscientists and soil experts. For instance, Bishwal (2017) cataloged a range of R packages applicable to the geoscience domain, and Sousa et al. (2020) enumerated a series of R packages suited for analyzing soil physical and hydraulic characteristics. These available packages are instrumental in augmenting R's analytical capabilities and flexibility in soil research field.

Reflecting a trend similar to that noted in ecological publications (Lai et al. 2019; Gao et al. 2025), the packages “lme4,” “vegan,” and “nlme” emerge as the most commonly utilized. Nevertheless, the disparity in their usage rankings suggests distinct preferences within the ecological versus soil science disciplines. In ecology, “lme4” is especially valued for its ability to handle complex data structures (Harrison 2014; Harrison et al. 2018; Lai et al. 2022), whereas soil science more frequently focuses on community‐level questions, for which the “vegan” package is well suited (Oksanen et al. 2024).

Furthermore, the open science movement emphasizes transparency and reproducibility in data and code sharing (NAS (National Academies of Sciences, Engineering, and Medicine), 2018; Elliott and Resnik 2019). R, being open‐source, provides a transparent and scriptable environment that enhances reproducibility (Grunsky 2002; Lai et al. 2019). This shift fosters collaboration and strengthens scientific rigor. At the same time, the number of specialized R packages tailored for soil science remains limited (Omuto and Gumbe 2009), and further development in this direction is encouraged—especially in response to increasing demands for big data analysis (Todd‐Brown et al. 2022).

Additionally, successful use of R requires a solid understanding of its statistical foundations (Webster 2001). Researchers should be encouraged not only to adopt R but also to strengthen their statistical literacy, ensuring that the growing ecosystem of packages is applied effectively and responsibly in soil science research. The results of this study indicate that there should be an active promotion of both the integration of R into research and the creation of R packages that are specifically crafted for the field of soil science.

Nonetheless, we acknowledge that this study has certain limitations. First, our analysis was restricted to 10 leading soil science journals, which may not fully represent the breadth of R usage in other relevant or regional publications. Second, we focused solely on R and did not conduct a systematic comparison with other emerging tools such as Python, which may also be gaining popularity in soil research. Future studies could expand the scope by incorporating a wider range of journals and comparative analyses of different statistical platforms.

Looking ahead, future research could explore the integration of R with other programming languages and data platforms to enhance reproducibility and analytical efficiency. There is also a growing need to develop and promote user‐friendly, domain‐specific R packages for soil scientists with limited coding experience. Moreover, with the increasing role of machine learning and spatial modeling in soil studies, future work could examine how advanced R‐based tools are adopted to address these emerging challenges.

## 5 Conclusions

The findings of this study clearly demonstrate the widespread and growing adoption of R in soil science research. With nearly a third of all articles analyzed relying on R as the primary data analysis tool, its significance in the field is undeniable. The consistent increase in R usage over the past decade, coupled with the identification of frequently used packages, such as “vegan,” “ggplot2,” “lme4,” “nlme,” and “randomForest,” highlights the diverse applications of R in addressing complex soil science questions. The variations in package usage across different journals reflect the specialized needs and research focuses within the discipline.

The increasing reliance on R suggests that soil science research is evolving towards more sophisticated and standardized analytical methods. The upward trend in the average number of R packages used per article indicates a progressive sophistication in analytical techniques within the soil science community. Moreover, this trend may foster greater transparency and reproducibility in research practices, aligning with the broader movement towards open science.

Moving forward, the continued expansion of the R ecosystem, especially in areas, such as big data analytics, geospatial modeling, and machine learning, is likely to play a crucial role in shaping the next generation of soil science research. Future studies could not only track these developments but also evaluate the effectiveness of emerging R‐based tools in improving the precision, scalability, and transparency of soil data analysis. This research underscores the importance of integrating cutting‐edge computational tools into soil science, which will continue to unlock deeper insights and foster interdisciplinary collaborations in the field.

## Author Contributions

**Meixiang Gao:** conceptualization (equal), data curation (lead), formal analysis (lead), investigation (equal), methodology (equal), resources (equal), software (lead), visualization (equal), writing – original draft (lead), writing – review and editing (lead). **Xiujuan Yan:** data curation (equal), formal analysis (lead), funding acquisition (equal), investigation (equal), methodology (equal), software (equal), visualization (equal), writing – original draft (equal), writing – review and editing (supporting). **Xin Li:** formal analysis (equal), investigation (equal), software (equal), writing – original draft (equal), writing – review and editing (supporting). **Ye Zheng:** formal analysis (equal), investigation (equal), software (equal), visualization (equal), writing – original draft (equal), writing – review and editing (supporting). **Jiangshan Lai:** conceptualization (lead), data curation (lead), formal analysis (lead), funding acquisition (equal), investigation (equal), methodology (lead), project administration (equal), software (lead), supervision (lead), visualization (equal), writing – original draft (equal), writing – review and editing (equal).

## Conflicts of Interest

The authors declare no conflicts of interest.

## Supporting information


Appendix S1.

Table S1.

Table S2.

## Data Availability

Data and codes have been provided as Appendix S1.
