# Supplementary material for: The Integration and Growth of R in Soil Research: A 10‐Year Analysis
Source: Ecol Evol. 2025 Jun 26;15(7):e71545. doi: 10.1002/ece3.71545 (PMC12202774; doi:10.1002/ece3.71545)
Supplement: Supplementary file 2 — Table S1. [file ECE3-15-e71545-s003.docx]

**Table S1:** Top 22 most frequently utilized software packages in the ten main journals of soil research, including Applied Soil Ecology, Biology and Fertility of Soils, Catena, European Journal of Soil Biology, European Journal of Soil Science, Geoderma, Pedosphere, Plant and Soil, Soil and Tillage Research, and Soil Biology and Biochemistry.

| Order | Package | Package Description | Maintainer and email | Release Date |
| --- | --- | --- | --- | --- |
| 1 | vegan | Community Ecology Package | Jari Oksanen, [jari.oksanen@oulu.fi](mailto:jari.oksanen@oulu.fi) | 2001-09-06 |
| 2 | ggplot2 | Create Elegant Data Visualisations Using the Grammar of Graphics | Thomas Lin Pedersen, thomas.pedersen@ posit.co | 2007-06-10 |
| 3 | lme4 | Linear Mixed-Effects Models using 'Eigen' and S4 | Ben Bolker, [bbolker+lme4@gmail.com](mailto:bbolker+lme4@gmail.com) | 2003-06-25 |
| 4 | nlme | Linear and Nonlinear Mixed Effects Models | R-core, [R-core@R-project.org](mailto:R-core@R-project.org) | 1999-11-23 |
| 5 | randomForest | Classification and regression based on a forest of trees using random inputs | Andy Liaw,  [andy_liaw@merck.com](mailto:andy_liaw@merck.com) | 2002-04-01 |
| 6 | multcomp | Simultaneous Inference in General Parametric Models | Torsten Hothorn, [Torsten.Hothorn@R-project.org](mailto:Torsten.Hothorn@R-project.org) | 2002-06-20 |
| 7 | agricolae | agricolae offers extensive functionality on experimental design especially for agricultural and plant breeding experiments, which can also be useful for other purposes. | Felipe de Mendiburu, [fmendiburu@lamolina.edu.pe](mailto:fmendiburu@lamolina.edu.pe) | 2007-01-25 |
| 8 | car | Companion to Applied Regression | John Fox  [jfox@mcmaster.ca](mailto:jfox@mcmaster.ca) | 2001-05-01 |
| 9 | emmeans | Estimated marginal means (EMMs) for many linear, generalized linear, and mixed models | Russell V. Lenth  [russell-lenth@uiowa.edu](mailto:russell-lenth@uiowa.edu) | 2017-11-05 |
| 10 | igraph | Routines for simple graphs and network analysis. It can handle large graphs very well and provides functions for generating random and regular graphs, graph visualization, centrality methods, and much more. | Kirill Müller,  [kirill@cynkra.com](mailto:kirill@cynkra.com) | 2005-01-25 |
| 11 | lavaan | Fit a variety of latent variable models, including confirmatory factor analysis, structural equation modeling, and latent growth curve models. | Yves Rosseel, Yves.Rosseel at UGent.be | 2012-09-08 |
| 12 | corrplot | Provides a visual exploratory tool on correlation matrix that supports automatic variable reordering to help detect hidden patterns among variables. | Taiyun Wei, [weitaiyun@gmail.com](mailto:weitaiyun@gmail.com) | 2010-04-10 |
| 13 | MASS | Support Functions and Datasets for Venables and Ripley's MASS | Brian Ripley, [ripley@stats.ox.ac.uk](mailto:ripley@stats.ox.ac.uk) | 2009-05-08 |
| 14 | phyloseq | phyloseq provides a set of classes and tools to facilitate the import, storage, analysis, and graphical display of microbiome census data. | Paul J. McMurdie, joey711 at gmail.com | 2013-04-22 |
| 15 | caret | Misc functions for training and plotting classification and regression models | Max Kuhn, mxkuhn at gmail.com | 2023-03-21 |
| 16 | ade4 | Analysis of Ecological Data : Exploratory and Euclidean Methods in Environmental Sciences | Aurélie Siberchicot, [aurelie.siberchicot@univ-lyon1.fr](mailto:aurelie.siberchicot@univ-lyon1.fr) | 2002-12-10 |
| 17 | picante | Integrating Phylogenies and Ecology, community phylogenetic and trait diversity | Steven W. Kembe, [steve.kembel@gmail.com](mailto:steve.kembel@gmail.com) | 2008-06-03 |
| 18 | psych | Functions are primarily for multivariate analysis and scale construction using factor analysis, principal component analysis, cluster analysis, and reliability analysis | William Revelle, revelle at northwestern.edu | 2024-06-27 |
| 19 | lmerTest | Tests in Linear Mixed Effects Models | Rune Haubo Bojesen Christensen, [Rune.Haubo@gmail.com](mailto:Rune.Haubo@gmail.com) | 2013-01-26 |
| 20 | Hmisc | Conversion of R objects to LaTeX and html code, recoding variables, caching, simplified parallel computing, encrypting and decrypting data using a safe workflow, general moving window statistical estimation, and assistance in interpreting principal component analysis. | Frank E Harrell Jr,  [fh@fharrell.com](mailto:fh@fharrell.com) | 2003-07-10 |
| 21 | MuMin | Multi-Model Inference | Kamil Bartoń, [kamil.barton@go2.pl](mailto:kamil.barton@go2.pl) | 2010-05-28 |
| 22 | plspm | Partial Least Squares Path Modeling (PLS-PM), Tenenhaus, Esposito Vinzi, Chatelin, Lauro， analysis for both metric and non-metric data, as well as REBUS analysis, Esposito Vinzi, Trinchera, Squillacciotti, and Tenenhaus | Frederic Bertrand, [frederic.bertrand@utt.fr](mailto:frederic.bertrand@utt.fr) | 2009-04-15 |
