# Supplementary material for: The Integration and Growth of R in Soil Research: A 10‐Year Analysis
Source: Ecol Evol. 2025 Jun 26;15(7):e71545. doi: 10.1002/ece3.71545 (PMC12202774; doi:10.1002/ece3.71545)
Supplement: Supplementary file 3 — Table S2. [file ECE3-15-e71545-s001.docx]

**Table S1:** R packages designed for soil science studies, sourced from ‘https://search.r-project.org/’ on September 8, 2024.

| Order | Package | Package Description | Maintainer and email | Release Date |
| --- | --- | --- | --- | --- |
| 1 | SWATmodel | The Soil & Water Assessment Tool is a small watershed to river basin-scale model used to simulate the quality and quantity of surface and ground water and predict the environmental impact of land use, land management practices, and climate change. | https://swat.tamu.edu/software/ | 1994 |
| 2 | sorcering | Soil Organic Carbon and CN Ratio Driven Nitrogen Modelling Framework | Marc Scherstjanoi, [marc.scherstjanoi@thuenen.de](mailto:marc.scherstjanoi@thuenen.de) | 2024-04-28 |
| 3 | Soil DB | Soil Database Interface | **Andrew Brown,** [andrew.g.brown@usda.gov](mailto:andrew.g.brown@usda.gov) | 2011-12-23 |
| 4 | HydroMe | Contains functions for estimating the parameters of infiltration and water retention mod els using the curve-fitting methods as discussed in Omuto and Gumbe (2009) | Christian Thine Omuto, [thineomuto@yahoo.com](mailto:thineomuto@yahoo.com) | 2013-05-04 |
| 5 | soilprofile | This package provides functions to graphically represent soil properties. | Gianluca Filippa,  https://www.github.com/null | 2013-08-23 |
| 6 | soiltexture | "The Soil Texture Wizard" is a set of R functions designed to produce texture triangles (also called texture plots, texture diagrams, texture ternary plots), classify and transform soil textures data. | Julien Moeys, [jules_m78-soiltexture@yahoo.fr](mailto:jules_m78-soiltexture@yahoo.fr) | 2017-03-29 |
| 7 | soilwater | Implementation of Parametric Formulas for Soil Water Retention or Conductivity Curve | Emanuele Cordano, [emanuele.cordano@gmail.com](mailto:emanuele.cordano@gmail.com) | 2017-07-29 |
| 8 | Ecohydmod | Ecohydrological Modelling | Rodolfo Souza, [rodolfomssouza@gmail.com](mailto:rodolfomssouza@gmail.com) | 2017-08-24 |
| 9 | aqp | Algorithms for Quantitative Pedology | Dylan Beaudette, [dylan.beaudette@usda.gov](mailto:dylan.beaudette@usda.gov) | 2018-12-29 |
| 10 | LWFBrook90R | Simulate Evapotranspiration and Soil Moisture with the SVAT Model LWF-Brook90 | Paul Schmidt-Walter, [paulsw@posteo.de](mailto:paulsw@posteo.de) | 2019-05-13 |
| 11 | TUWmodel | Lumped/Semi-Distributed Hydrological Model for Education Purposes | Alberto Viglione, [alberto.viglione@polito.it](mailto:alberto.viglione@polito.it) | 2020-02-26 |
| 12 | WCM | Water Cloud Model (WCM) for the Simulation of Leaf Area Index (LAI) and Soil Moisture (SM) from Microwave Backscattering | Ujjwal Singh, [ujjwalrsmt@gmail.com](mailto:ujjwalrsmt@gmail.com) | 2020-04-01 |
| 13 | spsh | Estimation and Prediction of Parameters of Various Soil Hydraulic Property Models | Tobias KD Weber, [tobias.weber@uni-hohenheim.de](mailto:tobias.weber@uni-hohenheim.de) | 2020-04-06 |
| 14 | Soil-app | Soil-app is a web application with a friendly click-point interface built through packages lodged in R software. The app is an advanced model of an open-source platform to support teaching and learning activities in soil analyses and fertilizer | Roberto Fritsche-Neto, [roberto.neto@usp.br](mailto:roberto.neto@usp.br) | 2020-11 |
| 15 | XPolaris | It represents an optimization of the Soil Survey Geographic database. This R package facilitates the access to large amounts of soil data within the US, currently stored online as raster images (TIFF). | Luiz Moro Rosso, [lhmrosso@ksu.edu](mailto:lhmrosso@ksu.edu) | 2021-09-23 |
| 16 | mpspline2 | Mass-Preserving Spline Functions for Soil Data | Lauren O'Brien, [obrlsoilau@gmail.com](mailto:obrlsoilau@gmail.com) | 2022-04-03 |
| 17 | SoilTesting | Organic Carbon and Plant Available Nutrient Contents in Soil | Bappa Das, [bappa.iari.1989@gmail.com](mailto:bappa.iari.1989@gmail.com) | 2022-04-14 |
| 18 | soilphysics | Soil Physical Analysis | Anderson Rodrigo da Silva, [anderson.agro@hotmail.com](mailto:anderson.agro@hotmail.com) | 2022-06-07 |
| 19 | pedometrics | Miscellaneous Pedometric Tools | Alessandro Samuel-Rosa, [alessandrosamuelrosa@gmail.com](mailto:alessandrosamuelrosa@gmail.com) | 2022-06-19 |
| 20 | soilhypfit | Modelling of Soil Water Retention and Hydraulic Conductivity Data | Andreas Papritz, [papritz@retired.ethz.ch](mailto:papritz@retired.ethz.ch) | 2022-08-31 |
| 21 | soiltestcor | A compilation of functions designed to assist users on the correlation analysis of crop yield and soil test values. Functions to estimate crop response patterns to soil nutrient availability and critical soil test values using various approaches | Adrian A. Correndo, [acorrend@uoguelph.ca](mailto:acorrend@uoguelph.ca) | 2022-12-06 |
| 22 | sharpshootR | A Soil Survey Toolkit | Dylan Beaudette, [dylan.beaudette@usda.gov](mailto:dylan.beaudette@usda.gov) | 2022-12-10 |
| 23 | soilHyp | Soil Hydraulic Properties | Ullrich Dettmann, [ullrich.dettmann@thuenen.de](mailto:ullrich.dettmann@thuenen.de) | 2023-02-02 |
| 24 | soilphysic | Basic and model-based soil physical analyses. | Anderson Rodrigo da Silva, [anderson.agro@hotmail.com](mailto:anderson.agro@hotmail.com); Anderson Rodrigo da Silva, [anderson.agro@hotmail.com](mailto:anderson.agro@hotmail.com); Adrian Bowman, [adrian.bowman@glasgow.ac.uk](mailto:adrian.bowman@glasgow.ac.uk); Adrian Bowman, [adrian.bowman@glasgow.ac.uk](mailto:adrian.bowman@glasgow.ac.uk) | 2023-02-07 |
| 25 | QI | Quantity-Intensity Relationship of Soil Potassium | Bappa Das, [bappa.iari.1989@gmail.com](mailto:bappa.iari.1989@gmail.com) | 2023-03-09 |
| 26 | soilchemistry | Computation of Properties Related to Soil Chemical Environment and Nutrient Availability | Bappa Das, [bappa.iari.1989@gmail.com](mailto:bappa.iari.1989@gmail.com) | 2023-03-21 |
| 27 | SQI | Soil Quality Index | Dr. Owais Ali Wani, [owaisaliwani@skuastkashmir.ac.in](mailto:owaisaliwani@skuastkashmir.ac.in) | 2023-04-10 |
| 28 | DMMF | Daily Based Morgan-Morgan-Finney (DMMF) Soil Erosion Model | Kwanghun Choi, [kwanghun.choi@yahoo.com](mailto:kwanghun.choi@yahoo.com) | 2023-04-26 |
| 29 | geomod | A Computer Program for Geotechnical Investigations | Festus Ngeno, [festus.k.ngeno@gmail.com](mailto:festus.k.ngeno@gmail.com) | 2023-05-05 |
| 30 | smosr | Acquire and Explore BEC-SMOS L4 Soil Moisture Data in R | Tatiana A. Shestakova, [tasha.work24@gmail.com](mailto:tasha.work24@gmail.com) | 2023-05-05 |
| 31 | soilfoodwebs | Soil Food Web Analysis | Robert Buchkowski, [robert.buchkowski@gmail.com](mailto:robert.buchkowski@gmail.com) | 2023-05-09 |
| 32 | soilassessment | Assessment Models for Agriculture Soil Conditions and Crop Suitability | Christian Thine Omuto, [thineomuto@yahoo.com](mailto:thineomuto@yahoo.com) | 2023-07-08 |
| 33 | washi | Washington Soil Health Initiative Branding | Jadey Ryan, [jryan@agr.wa.gov](mailto:jryan@agr.wa.gov) | 2023-09-07 |
| 34 | hwsdr | Interface to the 'HWSD' Web Services | Koen Hufkens, [koen.hufkens@gmail.com](mailto:koen.hufkens@gmail.com) | 2023-09-16 |
| 35 | ISRaD | Tools and Data for the International Soil Radiocarbon Database | Jeffrey Beem-Miller, [jbeem@bgc-jena.mpg.de](mailto:jbeem@bgc-jena.mpg.de) | 2023-09-21 |
| 36 | RPhosFate | Soil and Chemical Substance Emission and Transport Model | Gerold Hepp, gisler@ hepp.cc | 2023-10-07 |
| 37 | SoilR | Models of Soil Organic Matter Decomposition | Carlos A. Sierra, [csierra@bgc-jena.mpg.de](mailto:csierra@bgc-jena.mpg.de) | 2023-10-13 |
| 38 | simET | Tools for Simulation of Evapotranspiration of Field Crops and Soil Water Balance | Minguo Liu, [liumg15@lzu.edu.cn](mailto:liumg15@lzu.edu.cn>) | 2023-10-14 |
| 39 | OBIC | Calculate the Open Bodem Index (OBI) Score | Sven Verweij, [sven.verweij@nmi-agro.nl](mailto:sven.verweij@nmi-agro.nl) | 2023-10-18 |
| 40 | Rquefts | Quantitative Evaluation of the Native Fertility of Tropical Soils | Robert J. Hijmans, [r.hijmans@gmail.com](mailto:r.hijmans@gmail.com) | 2023-10-24 |
| 41 | ausplotsR | TERN AusPlots Australian Ecosystem Monitoring Data | Greg Guerin, [ggueri01@gmail.com](mailto:ggueri01@gmail.com) | 2023-11-17 |
| 42 | SoilTaxonomy | A System of Soil Classification for Making and Interpreting Soil Surveys | Andrew Brown, [andrew.g.brown@usda.gov](mailto:andrew.g.brown@usda.gov) | 2023-11-26 |
| 43 | febr | The model can be used to detect shifts in rates of evolution along branches. | Russell Dinnage, r.dinnage@gmail.com（ORCID） | 2024-01-27 |
| 44 | SoilConservation | Soil and Water Conservation | Dione Pereira Cardoso, [cardoso.dione@gmail.com](mailto:cardoso.dione@gmail.com) | 2024-04-28 |
| 45 | biologicalActivityIndices | Biological Activity Indices | Tanuj Misra, [tanujmisra102@gmail.com](mailto:tanujmisra102@gmail.com) | 2024-05-15 |
| 46 | SoilFunctionality | Soil Functionality Measurement | Tanuj Misra, [tanujmisra102@gmail.com](mailto:tanujmisra102@gmail.com) | 2024-05-23 |
| 47 | neonSoilFlux | Compute Soil Carbon Fluxes for the National Ecological Observatory Network Sites | John Zobitz, [zobitz@augsburg.edu](mailto:zobitz@augsburg.edu) | 2024-05-25 |
| 48 | SoilFDA | Fractal Dimension Analysis of Soil Particle Size Distribution | Fehim Jeelani Wani, [faheemwani@skuastkashmir.ac.in](mailto:faheemwani@skuastkashmir.ac.in) | 2024-05-27 |
| 49 | SoilSaltIndex | Soil Salinity Indices Generation using Satellite Data | Nobin Chandra Paul, [nobin.paul@icar.gov.in](mailto:nobin.paul@icar.gov.in) | 2024-06-11 |
| 50 | soiltestcorr | Soil Test Correlation and Calibration | Adrian A. Correndo, [acorrend@uoguelph.ca](mailto:acorrend@uoguelph.ca) | 2024-07-01 |
